# An aberrant fused in sarcoma liquid droplet of amyotrophic lateral sclerosis pathological variant, R495X, accelerates liquid–solid phase transition

**DOI:** 10.1038/s41598-024-59604-4

**Published:** 2024-04-17

**Authors:** Yutaro Shiramasa, Ryu Yamamoto, Norika Kashiwagi, Fuka Sasaki, Sawaka Imai, Mikihito Ike, Soichiro Kitazawa, Tomoshi Kameda, Ryo Kitahara

**Affiliations:** 1 Graduate School of Pharmacy, Ritsumeikan University, 1-1-1 Nojihigashi, Kusatsu, Shiga 525-8577 Japan; 2 College of Pharmaceutical Sciences, Ritsumeikan University, 1-1-1 Nojihigashi, Kusatsu, Shiga 525-8577 Japan; 3 Artificial Intelligence Research Center, National Institute of Advanced Industrial Science and Technology (AIST), 2-3-26, Aomi, Koto-ku, Tokyo, 135-0064 Japan

**Keywords:** Intrinsically disordered proteins, Molecular biophysics, Protein aggregation, Protein aggregation

## Abstract

Intracellular aggregation of fused in sarcoma (FUS) is associated with the pathogenesis of familial amyotrophic lateral sclerosis (ALS). Under stress, FUS forms liquid droplets via liquid–liquid phase separation (LLPS). Two types of wild-type FUS LLPS exist in equilibrium: low-pressure LLPS (LP-LLPS) and high-pressure LLPS (HP-LLPS); the former dominates below 2 kbar and the latter over 2 kbar. Although several disease-type FUS variants have been identified, the molecular mechanism underlying accelerated cytoplasmic granule formation in ALS patients remains poorly understood. Herein, we report the reversible formation of the two LLPS states and the irreversible liquid–solid transition, namely droplet aging, of the ALS patient-type FUS variant R495X using fluorescence microscopy and ultraviolet–visible absorption spectroscopy combined with perturbations in pressure and temperature. Liquid-to-solid phase transition was accelerated in the HP-LLPS of R495X than in the wild-type variant; arginine slowed the aging of droplets at atmospheric conditions by inhibiting the formation of HP-LLPS more selectively compared to that of LP-LLPS. Our findings provide new insight into the mechanism by which R495X readily forms cytoplasmic aggregates. Targeting the aberrantly formed liquid droplets (the HP-LLPS state) of proteins with minimal impact on physiological functions could be a novel therapeutic strategy for LLPS-mediated protein diseases.

## Introduction

Recently, with the discovery of membraneless organelles by protein self-assembly, known as biomolecular liquid–liquid phase separation (LLPS)^1–5^, there has been an increase in interest in the mechanism of LLPS formation and the nexus of LLPS and protein aggregation, such as misfolded proteins, amyloid fibrils, and amorphous structures^6–9^. Accumulation of misfolded aggregation-prone proteins including small oligomeric species and misfolded species and amyloid fibrils in neurons can be cytotoxic^3^. However, effective therapeutics targeting these protein aggregates are still in development for many neurodegenerative diseases. Various intrinsically disordered proteins and proteins with intrinsically disordered regions, including nucleoproteins, reportedly produce LLPS as protein self-assembly is triggered by cellular stresses, such as oxidative stress or supersaturation of the protein caused by external stimuli such as pH, salt, temperature, and pressure^3,4,10^. Generally, liquid condensates (e.g., droplets) exhibit liquid-like properties, characterized by fusion and rapid molecular rearrangement. Over time, their properties change from those of a liquid to those of a solid; this process is known as droplet aging^4,11^. To understand the entire protein aggregation process and develop new drugs for neurodegenerative diseases, it is important to investigate liquid droplet formation and liquid–solid phase transition. The structure and material properties of liquid droplets and aggregates have been studied using various biophysical techniques, including ultraviolet–visible absorption (UV–Vis) spectroscopy^10^, electron microscopy^12^, fluorescence recovery after photobleaching (FRAP)^13,14^, fluorescence correlation spectroscopy^2^, single-molecule Förster resonance electron transfer^15^, microrheology^16^, Raman microscopy^8,17^, atomic force microscopy^9^, electron paramagnetic resonance spectroscopy^18^, and nuclear magnetic resonance spectroscopy^18–21^.

Fused in sarcoma (FUS) is an RNA-binding protein involved in RNA processing, transport, and translation. It is intrinsically disordered except for the RNA recognition motif and the zinc finger region^22,23^. FUS dysfunction has been linked to amyotrophic lateral sclerosis (ALS) and frontotemporal lobar degeneration. In particular, intracellular aggregation of this protein has been implicated in the onset of ALS^24^. The majority of the mutations in *fus* associated with ALS are located in the C-terminal region, which includes the Arg-Gly Gly (RGG) domains and nuclear localization signaling (NLS)^22,23^. Such mutations would disrupt the interactions between FUS-NLS and transportin, which translocates FUS from the cytoplasm to the nucleus, leading to cytoplasmic accumulation and aberrant aggregation of FUS^1^. Under stress conditions, FUS is also concentrated in cytoplasmic stress granules^25^.

The FUS wild-type (WT) can form at least two types of LLPS, low-pressure LLPS (LP-LLPS) and high-pressure LLPS (HP-LLPS)^26^, as pressure and temperature vary. The former dominates below 2 kbar and the latter above 2 kbar. Even under varying atmospheric conditions, both LLPS states may coexist. To date, several studies on the LP-LLPS state of FUS at atmospheric pressure have been conducted. The LP-LLPS state is primarily mediated by multivalent cation-π interactions between Tyr residues in the low-complexity (LC) region and Arg residues in the RGG regions^14^. Furthermore, according to FRAP measurements, LP-LLPS loses its liquidity within several hours via the aging process^27,28^. Different LLPS states have been observed for several RNA-binding proteins, including FUS and TDP-43, at both high (2 M ~) and low salt concentrations (~ 0.2 M). In contrast to low-salt-mediated LLPS, which is primarily stabilized by multivalent cation-π interactions, high-salt-mediated LLPS is considered to be stabilized by hydrophobic and non-ionic interactions^29^.

We have previously investigated the HP-LLPS state of FUS-WT under various pressure and temperature conditions using high-pressure UV–Vis spectroscopy and high-pressure microscopy^26,28,30^. The HP-LLPS state was more condensed, had lower susceptibility to dissolution by 1,6-hexanediol, and had a smaller partial molar volume than LP-LLPS^26^. Molecular dynamics (MD) simulations showed that electrostatic interactions were destabilized, whereas cation-π, π–π, and hydrophobic interactions were stabilized in HP-LLPS^26^. In addition, pressure-jump UV–Vis experiments revealed that the formation and disappearance rates of HP-LLPS were ~ 2- and ~ 20-fold slower than those of LP-LLPS, respectively. The slower disappearance rate suggests that the intermolecular interactions in HP-LLPS were stronger than those in LP-LLPS^30^. In fact, HP-LLPS accelerated liquid–solid phase transition^26,28^. These results indicate that the formation of HP-LLPS via 1-phase or LP-LLPS is a physiological pathway that leads to the irreversible formation of solid aggregates. We also found that HP-LLPS formation was suppressed by arginine, dopamine, and pyrocatechol, suggesting that these small molecules may be potential drug candidates for ALS-causing FUS^28^.

In addition, various FUS mutations have been reported in patients with ALS (e.g., G156E, P525L, and R495X)^22,27,31,32^. R495X has a premature stop codon at position 495 and does not have an NLS region, resulting in the loss of nuclear localization. As a result, R495X preferentially accumulates in neuronal and non-neuronal cells and forms cytoplasmic granules^33^. In fact, R495X is found in patients including those with juvenile-onset familial and with sporadic ALS, who develop a more aggressive disease phenotype^31,34,35^. However, the molecular mechanism underlying accelerated cytoplasmic granule formation in patients with ALS remains poorly understood. Here, we report the differences in the physicochemical properties of FUS-WT and R495X, including the cloud points at different protein concentrations, the pressure–temperature (*p–T*) phase diagram, the formation and disappearance rates, and the phase transition to the solid aggregate of their LLPS states. The effects of arginine, dopamine, and pyrocatechol on droplets were investigated. Furthermore, MD simulations were performed to study the backbone dynamics and intramolecular interactions of the proteins.

## Results

### Concentration dependence of the cloud point

**Figure 1:**
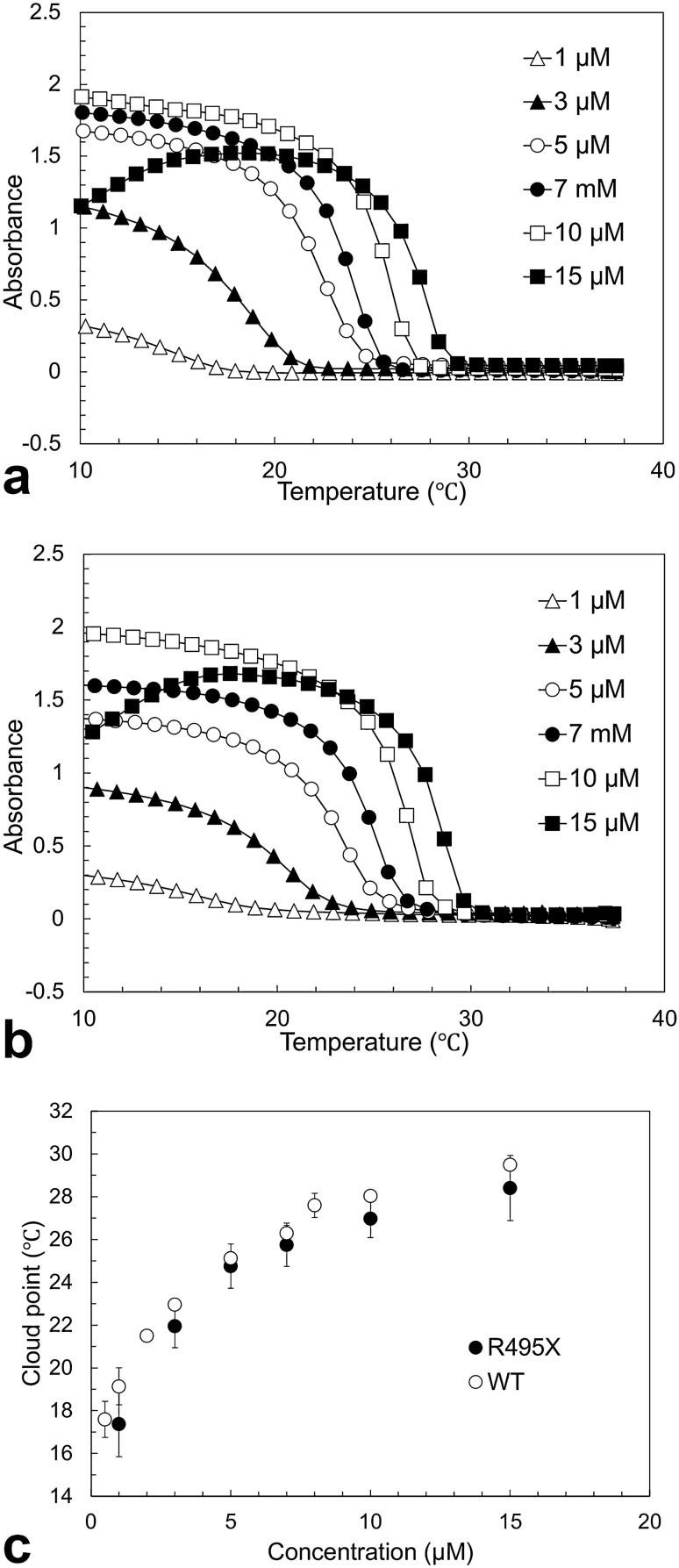
Temperature-scan experiments of FUS-R495X and -WT. (**a**) Changes in absorbance (i.e., turbidity) for FUS-R495X as temperature decreases from 38 to 10 °C at 1 bar. (**b**) Changes in absorbance for FUS-WT as temperature decreases from 38 to 10 °C at 1 bar. (**c**) The cloud points of FUS-WT (open circles) and FUS-R495X (closed circles) at different protein concentrations at 1 bar. Mean and deviation of the cloud points are shown in the panel. Temperature-scan experiments were performed three times for R495X and five times for WT. Representative data sets are shown in the panels (**a**) and (**b**).

Figure 1 shows the concentration dependence of the cloud point, i.e., the formation temperature of LLPS in the protein solution. Absorbance, i.e., turbidity, with temperature change for FUS R495X and WT, is plotted in Fig. 1a and b, respectively. The increase in absorbance with cooling indicated LP-LLPS formation, signifying an exothermic reaction. The absorbance increased with increasing protein concentration, likely indicating an increase in the number of droplets. However, when the protein was at 15 μM, a temperature-dependent decrease in absorbance with cooling was observed below 20 °C. It is possible that precipitation of the protein occurred because of droplet clustering or amorphous aggregation in the sample chamber. Figure 1c shows the concentration dependence of the cloud points for WT and R495X. The standard deviation of the cloud point in multiple experiments (e.g., 3–5 times) was within 0.9 °C for WT and 1.5 °C for R495X over the entire concentration range. Since the measurement error does not depend on protein concentration, it appears that the protein precipitation has not yet occurred near the cloud point. Both protein solutions showed similar upper critical solution temperature phase behaviors, indicating that protein–protein and protein–solvent interactions (e.g., the Flory–Huggins parameter) would be similar between both protein solutions at 1 bar, where LP-LLPS is dominant.

### Pressure–temperature phase diagram

**Figure 2:**
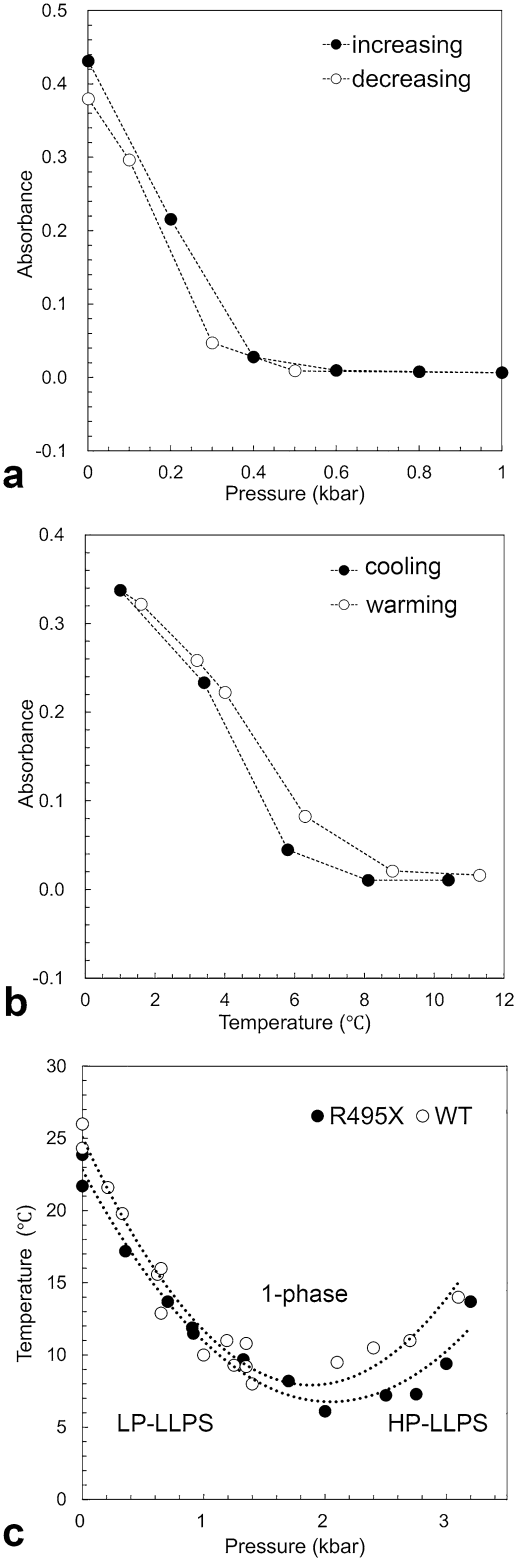
Pressure and temperature dependence of FUS-R495X liquid–liquid phase separation (LLPS). (**a**) Changes in absorbance with increasing (closed circles) and decreasing (open circles) pressures at 17.2 °C (left). (**b**) Changes in absorbance with cooling (closed circles) and warming (open circles) at 2.75 kbar (left). (**c**) Pressure and temperature phase diagram of LLPS. The cloud points and transition pressures were obtained for 5 μM of FUS-R495X (closed circles). FUS-WT data (open circles) are reproduced from the previous report^26^. The phase diagram was generated by multiple temperature- and pressure-scan experiments for 5 μM of the proteins. These experiments were conducted once under each condition.

The *p*–*T* phase diagram of LLPS provides basic knowledge for understanding the behavior of the protein solution. It allows for predicting a pressure–temperature combination at which a protein solution of a given concentration will phase-separate^10,26^. Temperature-scan experiments at different pressures and pressure-scan experiments at different temperatures were performed on R495X to determine the cloud points and transition pressures, respectively. Figure 2a and b show the representative pressure- and temperature-scan experiments of R495X, respectively. For comparison, WT data of the representative pressure- and temperature-scan experiments are shown in Fig. S1a and b, respectively. The turbidity of the solution changed reversibly with variations in pressure and temperature. Only the reversible cloud points and transition pressures were estimated, resulting in a *p*–*T* phase diagram for the LLPS of R495X (Fig. 2c). The *p*–*T* phase diagram indicates the two types of LLPS states: LP-LLPS, stable at < 2 kbar, and HP-LLPS, stable at > 2 kbar, each with a different partial molar volume. If the slope of the LP-LLPS-1-phase curve below 2 kbar is negative and the slope of the 1-phase-HP-LLPS curve above 2 kbar is positive in the *p–T* phase diagram, the partial molar volume should decrease in the order of LP-LLPS, 1-phase, and HP-LLPS, indicating that HP-LLPS is more condensed than LP-LLPS. Most importantly, LP-LLPS appears to change continuously into HP-LLPS as the pressure increases under low-temperature conditions, suggesting that HP-LLPS is rare but exists with LP-LLPS in equilibrium even under atmospheric conditions. Comparison of the *p*–*T* phase diagrams for R495X and WT^26^ revealed that the cloud points of R495X were similar to those of WT over the examined pressure range.

### Progressive increase in the intra- and inter-molecular interactions within the droplet

**Figure 3:**
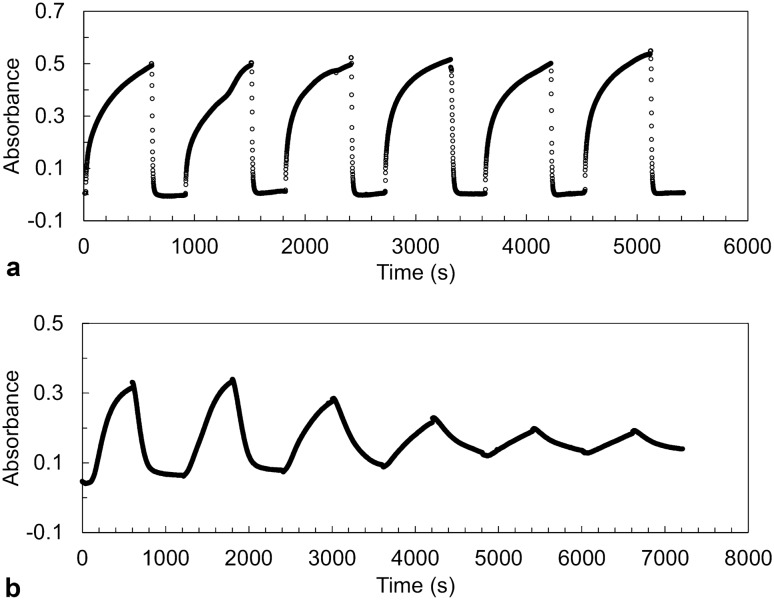
Pressure-jump relaxation study of FUS-R495X. (**a**) Changes in absorbance of R495X (5 μM) with six pressure cycles between 1 bar and 1.2 kbar at 9.5 °C. (**b**) Changes in absorbance of R495X (5 μM) with six pressure cycles between 2.0 and 3.5 kbar at 9.4 °C.

Using high-pressure UV–Vis spectroscopy, we investigated the pressure-jump kinetics of the two LLPS states of R495X, as shown in Fig. 3. Figure 3a shows the time course of the absorbance when the pressure was varied between 1 bar and 1.2 kbar every 10 min at 9.5 °C, where the LP-LLPS state and the homogeneous phase are the most stable, respectively. On the one hand, when the pressure decreased from 1.2 kbar to 1 bar, the absorbance increased, indicating LP-LLPS formation. On the other hand, when the pressure increased to 1.2 kbar, the absorbance rapidly decreased to zero, indicating LP-LLPS disappearance. Therefore, the formation and disappearance of LP-LLPS were reproduced reversibly with decreasing and increasing pressure, respectively. Figure 3b shows the time course of the absorbance when the pressure is changed between 2 and 3.5 kbar at 9.4 °C, where the homogeneous phase and the HP-LLPS state are most stable, respectively. Notably, HP-LLPS formation and disappearance were incomplete within 10 min when the pressure was repeatedly changed. These results indicate that irreversible protein aggregate formation, namely progressive increase in the intra- and inter-molecular interactions, is more pronounced for HP-LLPS than for LP-LLPS.

To quantify the formation kinetics of the two LLPS states of R495X, absorption profiles were analyzed using the Jonson–Mehl–Avrami–Komogorov (JMAK) function (Eq. 1)^30,36–38^, widely used in crystallography to analyze first-order phase transition kinetics. Assuming nucleation and growth mechanisms, the transformed ratio *X*(*t*) can be expressed by Eq. (1) without considering the overlap between different growing regions.

1 $$X\left( t \right) = V\left( t \right)/V = 1 - {\text{exp}}\left( { - \left( {kt} \right)^{n} } \right)$$

where *V*(*t*) is the time-dependent volume fraction, *V* is the total volume fraction, *k* is the reaction rate constant, and *n* is the Avrami exponent. The JMAK theory is applicable to the early and intermediate stages of the reaction, where the overlap between different domains can be ignored.

Figure S2a shows the time-dependent absorbance of R495X at 1 bar after a pressure jump from 1.2 kbar, indicating the formation of LP-LLPS. The absorbance was normalized to the endpoint absorbance (i.e., 600 s), corresponding to *X*(*t*). Based on the double logarithmic plot (Fig. S2b), i.e., ln *t* vs. ln [− ln(1 − *X*(*t*))], the values of *k* and *n* were obtained from the intercept and slope of the linear regression of the plot, respectively. The methodological details of this analysis are discussed in the literature^30^. The changes in *n,* along with the transformed fraction, are shown in Fig. S2c. The values of *n* were greater than 3 at the initial stage of the reaction and then decreased rapidly to approximately 0.5 at the intermediate stage. The values of *k*, *n*, and *t*_1/2_ for formation and *t*_1/2_ for disappearance are summarized in Table 1 as averages of six pressure cycles. Note that the *n* value was obtained as an average of the transformed fractions between 0.2 and 0.6. Figure S2d shows the time-dependent absorbance at 3.5 kbar after a pressure increase from 2 kbar, indicating the formation of HP-LLPS. A same analysis (i.e., ln *t* vs. ln [− ln(1 − *X*(*t*))] plot) was performed (Fig. S2e and f), and the values of *k*, *n*, and *t*_1/2_ for the formation and *t*_1/2_ for the disappearance of HP-LLPS are listed in Table 1. As the irreversible process in HP-LLPS progressed, the values of *t*_1/2_ for formation and disappearance gradually increased (Fig. 3). On average, the *t*_1/2_ values for HP-LLPS formation and disappearance were approximately 5- and 26-fold longer than those for LP-LLPS. Particularly, the slower disappearance of HP-LLPS than that of LP-LLPS indicates that HP-LLPS has stronger intermolecular interactions between polypeptide chains. As predicted by previous MD simulations in the case of WT, cation-π, non-ionic, and hydrophobic interactions are stronger, whereas electrostatic interactions are weaker in HP-LLPS than in LP-LLPS^26^. In addition, solvent-separated hydrophobic interactions were predicted to occur under high pressure in HP-LLPS^10^. These stronger and solvent-separated hydrophobic interactions could lead to denser molecular packing in HP-LLPS than that in LP-LLPS of R495X.

**Table 1:** Kinetic parameters obtained using the Johnson–Mehl–Avrami–Kolmogorov (JMAK) theory.

	*n*^a,b^	*k*^b^ (h^−1^)	*t*_1/2_^b^ (s)
LP-LLPS			
Formation	0.62 ± 0.03	31 ± 9	60 ± 30
Vanishing			6 ± 2
HP-LLPS			
Formation	2.1 ± 0.3	11 ± 2	280 ± 50
Vanishing			160 ± 50

^a^Avrami exponent. ^b^Error and standard deviation.

As the JMAK theory is applied to crystal formation, the Avrami exponent *n* can be coupled with the nucleation and growth of crystals using the following Eq. (2):

2 $$n = n_{1} + dn_{2}$$

where *n*_1_ = 1 for constant nucleation and 0 in the absence of nucleation, *d* is the growth dimension, *n*_2_ = 1 for interface-controlled growth, and *n*_2_ = 1/2 for diffusion-controlled growth^38^. Moreover, *n* = 2.5 is assumed for constant nucleation and spherical diffusion-controlled growth. Values of *n* < 1 were attributed to decreased nucleation and growth rates, a situation that can be observed in the later stages of crystal formation when the diffusion zones of different crystals begin to overlap. When droplet formation is accompanied by spherical growth, *n* = 2–3 in the early stages of LP- and HP-LLPS formation signifies constant nucleation with diffusion-controlled growth or the absence of nucleation with interface-controlled growth (i.e., growth from preexisting nuclei). Instead, *n* = 0.6 at the middle stage of LP-LLPS formation can be attributed to decreasing nucleation and growth rates with diffusion-controlled growth. Although *n* = 1.5–2 at the middle stage of HP-LLPS formation could be attributed to the decreasing nucleation and constant growth rates with diffusion-controlled growth, a gradual decrease in excess solute in the sample solution should result in decreased nucleation and growth rates. A large increase in *n* values was observed at the end of the formation process. Such a large increase in *n* can be associated with the loss of the amorphous phase at the end of the crystallization process^39^. Finally, the kinetic parameters of R495X LLPS were compared with those of WT LLPS. The *t*_1/2_ values for LP-LLPS and HP-LLPS formation and disappearance were ~ threefold longer in R495X than in the WT^30^. This could be attributed to R495X having stronger intermolecular interactions than the WT.

### Liquid–solid phase transition

**Figure 4:**
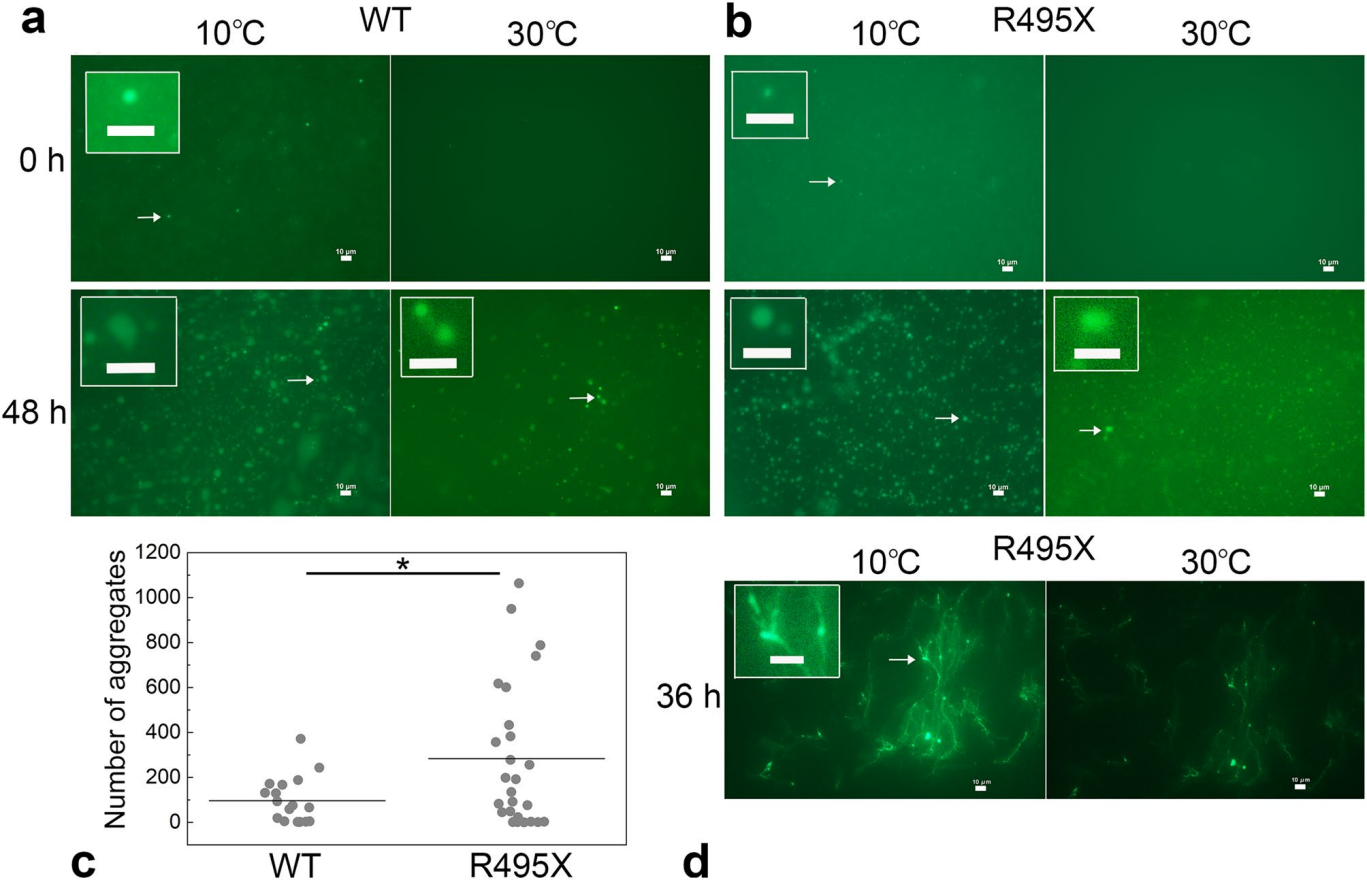
Generation of solid aggregates of FUS-WT and R495X. Time-dependent changes in fluorescence microscope images were investigated for liquid and solid condensates of WT (**a**) and R495X (**b**) (5 μM) incubated at 10 °C (left) and when the sample temperature was increased to 30 °C after incubation at 10 °C (right). Fluorescence microscope experiments were performed in triplicate for each experimental condition. (**c**) The number of aggregates of WT and R495X that did not disappear when heated to 30 °C after 48 h of incubation at 10 °C. The data of WT and R495X were obtained from 18 and 26 microscopic images, respectively. Means of particle counts are shown by lines. *0.01 < *P* < 0.05 using Student’s t-test. (**d**) Fluorescence microscope images of R495X after 36 h incubation at 10 °C (left) and when the sample temperature was increased to 30 °C (right). Data from a different sample from (**b**). Scale bar = 10 µm. The contrast and brightness of the images were adjusted using Adobe Photoshop 2023 (Adobe, Mountain View, CA, USA).

Liquid droplets generally exhibit liquid-like properties initially; however, as they age, they can transform into a more solid-like state, such as gels or amyloid- and non-amyloid-like aggregates. In this study, the progressive increase in the intra- and inter-molecular interactions that contribute to droplet aging was accelerated by HP-LLPS and was more pronounced in R495X than in the WT^30^. To further investigate the droplet aging of the proteins, microscopic observations were performed on the protein condensates. After sample preparation of WT and R495X containing 5% green fluorescence protein (GFP)-fused FUS-WT, undesired aggregates were removed by centrifugation. Notably, in vitro and *in cellula* experiments have shown that GFP-FUS also forms liquid droplets^14,26^. Fluorescence microscopy images were obtained as the temperature was decreased to 10 °C. Many small protein condensates were observed in the sample chamber. Figure 4a and b depict the microscope images of WT and R495X, respectively, captured after 0 and 48 h of incubation at 10 °C. When the microscope images were captured immediately after condensate formation, i.e., 0 h, several condensates were observed in R495X as well as WT, indicating that GFP-FUS-WT was incorporated into the R495X condensates. When the temperature of the variable temperature plate was increased above the cloud point, e.g., 30 °C (right panels), the condensates rapidly disappeared, indicating that they retained their liquid-like property, i.e., liquid droplets. After 24 h of incubation at 10 °C, several spherical and nonspherical droplets with strong fluorescence intensities were observed for WT and R495X at the bottom of the sample chamber. Notably, some did not disappear completely when the temperature was increased to 30 °C, indicating that liquid droplets developed irreversible properties. Irreversible aggregates accumulated at the bottom of the chamber as incubation time increased. After 48 h of incubation at 10 °C, the number of aggregates that did not disappear when heated to 30 °C increased markedly (Fig. 4a,b). The amount of irreversible aggregates was higher for R495X than for WT, as shown in Fig. 4c. During multiple experiments, fibrous aggregates growing in a reticulated pattern were also observed in R495X after an incubation period of 24 h or longer (Fig. 4d, left). Some spherical aggregates appear to be interconnected by fibers. These fibrous aggregates also exhibited an irreversible response to temperature changes (Fig. 4d, right). Similar fibrous products were also observed in the WT, albeit in smaller amounts (Fig. S3). Moreover, repetition of the experiment revealed a variation in the number of aggregates.

**Figure 5:**
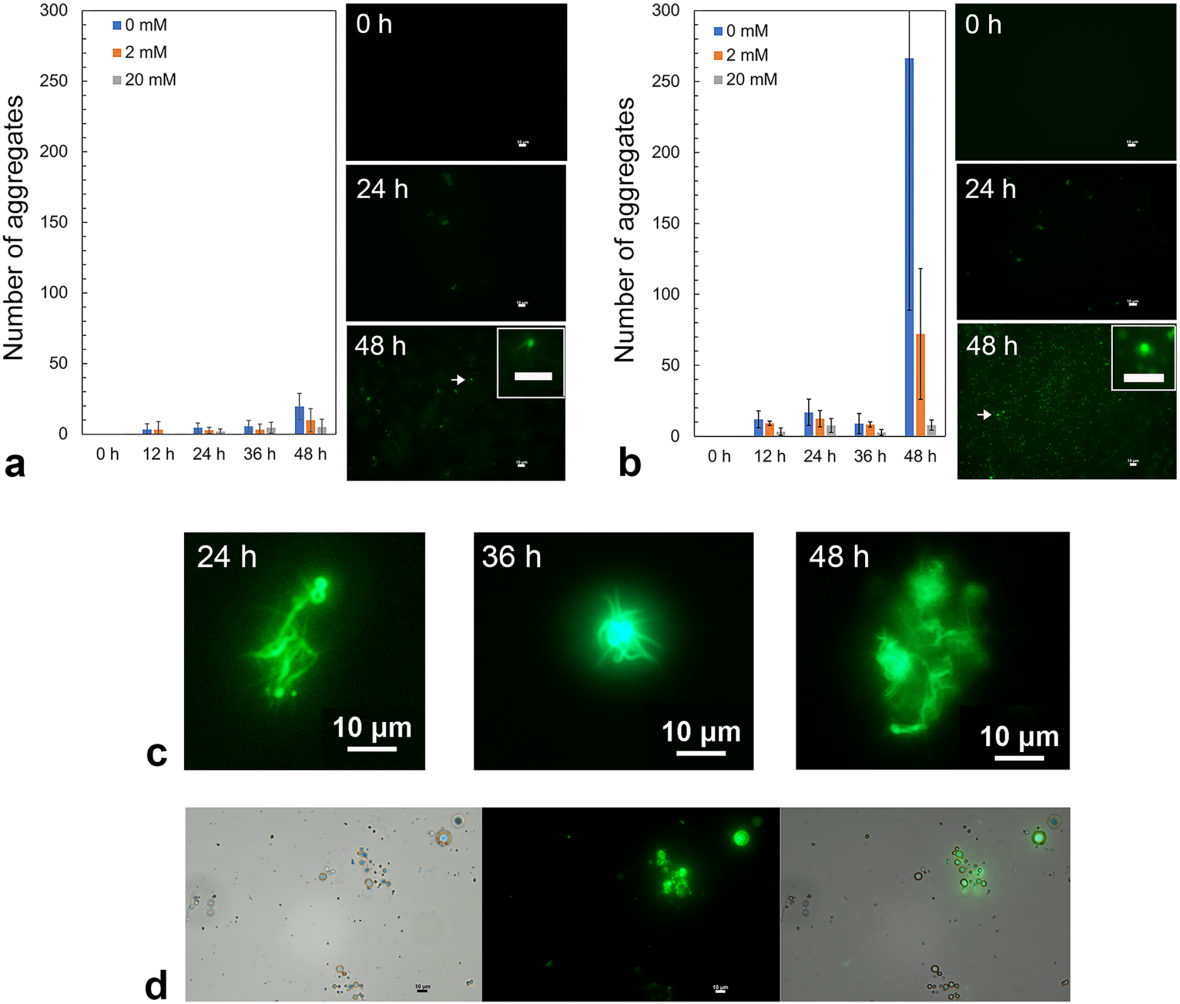
Thioflavin T assay of solid aggregates of FUS-WT and R495X. (**a**) Time-dependent changes in the number of FUS-WT aggregates at 0, 2, and 20 mM arginine concentrations in the buffer solution (left). Fluorescence microscope images collected at 0 h, 24 h, and 48 h incubation at 10 °C (right). An expanded image of the aggregate indicated by an arrow is shown in the inset. Images were collected from two experiments. (**b**) Time-dependent changes in the number of FUS-R495X aggregates at 0, 2, and 20 mM arginine concentrations in the buffer solution (left). Fluorescence microscope images collected at 0 h, 24 h, and 48 h incubation at 10 °C (right). An expanded image of the aggregate indicated by an arrow is shown in the inset. Images were collected from one experiment. (**c**) Fibrous aggregates of R495X observed when heated to 30 °C after 24 h, 36 h, or 48 h of incubation at 10 °C. (**d**) Comparison of bright-field (left) and fluorescence (middle) microscope images of WT condensates after 36 h incubation at room temperature (20–22 °C); their merged image is shown on the right. White or black scale bar = 10 mm.

Thioflavin T (ThT) assays using fluorescence microscopy were also performed after 12, 24, 36, and 48 h of incubation time at 10 °C to obtain structural information on the protein aggregates (Fig. 5a,b). Note that FUS without GFP fusion was used in the ThT assays. The fluorescence intensity from ThT bound to the protein aggregates became stronger with time in the droplets of both WT and R495X, indicating the growth of an intermolecular β-sheet structure, namely aging, in the droplets. Fibers extending from the spherical aggregates and clusters of multiple aggregates were also observed in the proteins (Figs. 5c and S3b). The degree of formation of the β-sheet structure seems to vary among the droplets (Fig. 5d).

### Inhibitory effect of low molecular weight compounds on LLPS formation and liquid–solid phase separation

**Figure 6:**
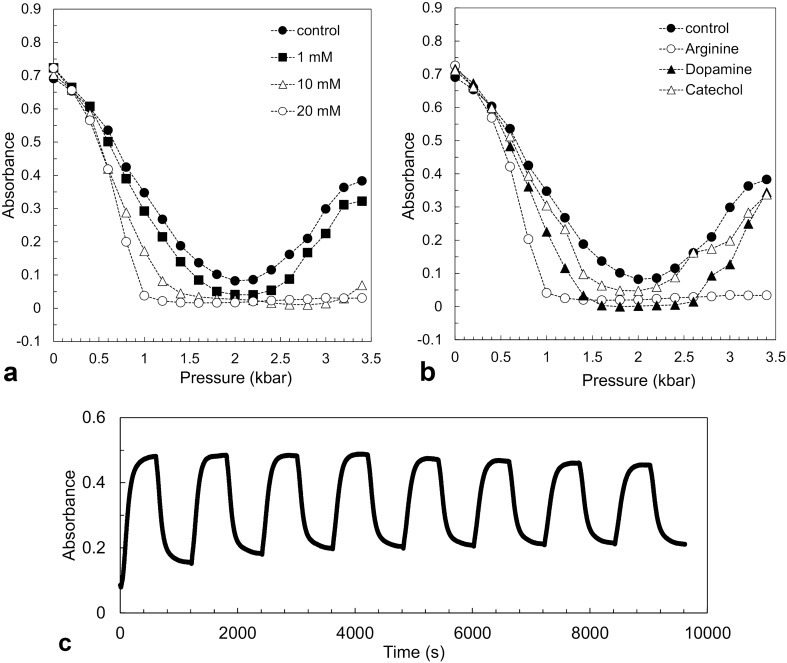
Effects of small compounds on the formation of FUS-R495X liquid–liquid phase separation (LLPS). (**a**) Pressure-dependent changes in absorbance of R495X (5 μM) with different arginine concentrations at 9.5 °C. (**b**) Pressure-dependent changes in absorbance of R495X (5 μM) with 20 mM of arginine, dopamine, and pyrocatechol at 9.5 °C. (**c**) Changes in absorbance of R495X (5 μM) with 1 mM of arginine with eight pressure cycles between 2.0 and 3.5 kbar at 9.3 °C.

In FUS-WT, we have previously found that 20 mM arginine, dopamine, and pyrocatechol suppressed the formation of HP-LLPS more than that of LP-LLPS^28^. In the present study, we examined the effects of these three compounds on LLPS formation in R495X. Figure 6a shows the effect of arginine on LP- and HP-LLPS formation at 9.5 °C. With arginine concentrations increasing up to 20 mM, absorbance on the low-pressure side decreased slightly, whereas that on the high-pressure side decreased significantly. These behaviors along the transition curve indicate that the effect of arginine on HP-LLPS is more significant than that on LP-LLPS. Arginine suppressed the formation of HP-LLPS in both WT^28^ and R495X. Figure 6b shows the effects of dopamine and pyrocatechol on LLPS formation. At 20 mM, these compounds showed a weaker inhibitory effect on the HP-LLPS of R495X than that of arginine.

The effect of arginine on the irreversible properties of HP-LLPS, i.e., aging and the formation of solid aggregates, was also investigated. Pressure-jump experiments were performed to confirm whether arginine suppresses the irreversible properties of HP-LLPS. The absorbance change was irreversible and did not recover even after three cycles in the absence of arginine (Fig. 3). Unexpectedly, even 1 mM arginine largely suppressed these irreversible properties, as shown in Fig. 6c. Even after repeating the eight pressure cycles, the change in the absorbance remained at 60% of the initial value.

**Figure 7:**
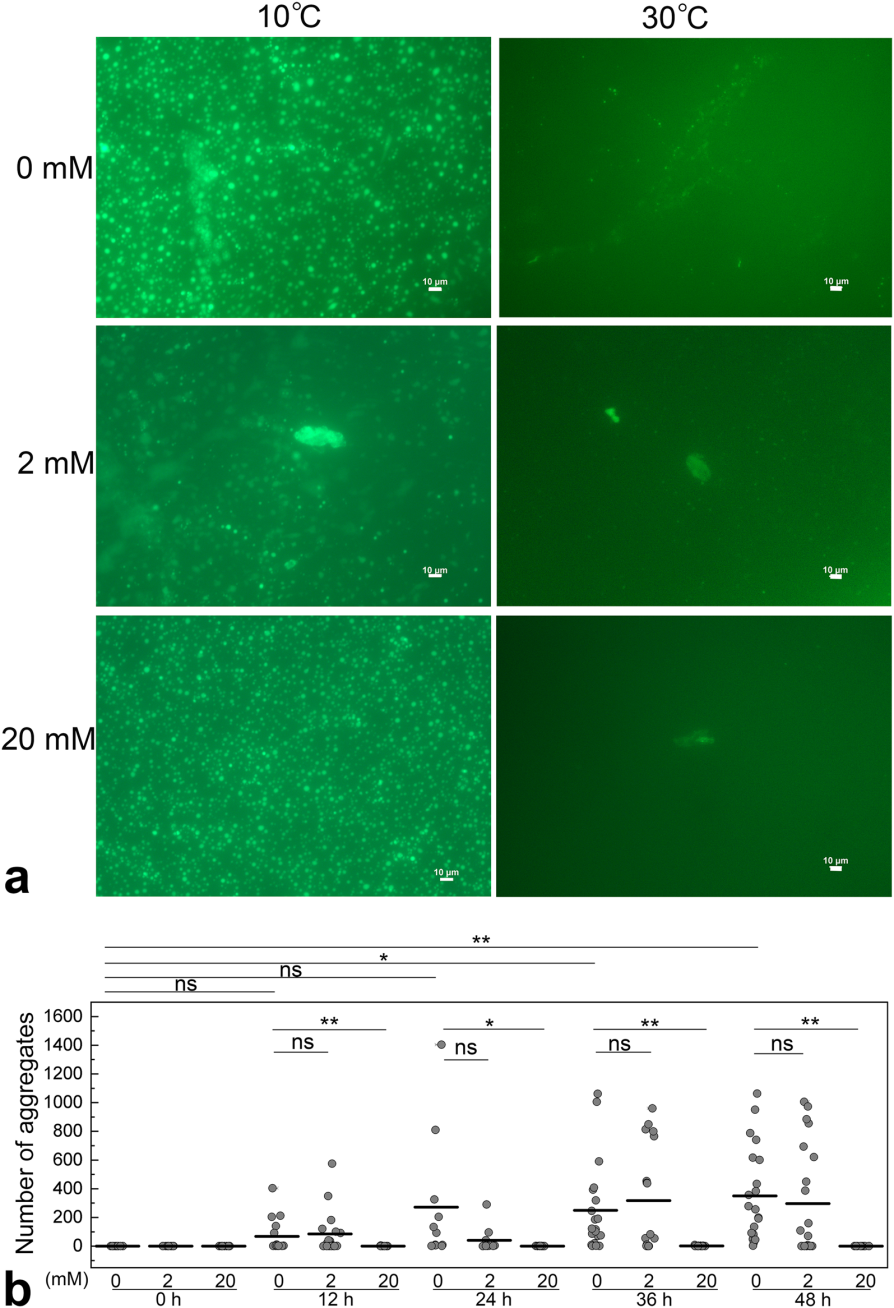
Arginine extends the reversible property of R495X droplets. (**a**) Fluorescence microscope images of R495X (5 μM) with different arginine concentrations (0–20 mM) were obtained at 10 °C (left) and 30 °C (right) after 36 h incubation at 10 °C. Scale bar = 10 µm. The contrast and brightness of the images obtained at 30 °C were adjusted using Adobe Photoshop 2023 (Adobe, Mountain View, CA, USA). (**b**) The number of remaining droplets when heated to 30 °C after 0–48 h of incubation at 10 °C. Fluorescence microscope experiments were performed in triplicate for each experimental condition. Means of particle counts were calculated using 10–27 microscopic images for each condition. *P < 0.05, **P < 0.01, and ns = not significant using Student’s t-test.

In addition, fluorescence microscopy observations in solutions containing 2 and 20 mM arginine were also performed for R495X. Figure 7a shows microscope images of R495X with different arginine concentrations (0, 2, and 20 mM) after 36 h of incubation at 10 °C (Fig. 7a, left). Droplets formed at 10 °C, regardless of arginine concentration. The right column shows the chamber interior when heated to 30 °C. If the droplets retain their liquid state, they should disappear reversibly at 30 °C, where the 1-phase is the most stable phase. When the arginine concentration was 0 mM, the number of remaining droplets with heating increased over time, indicating that aging had progressed in the droplets. However, the number of remaining droplets decreased at an arginine concentration of 20 mM. When the arginine concentration was 2 mM, the aggregation inhibiting effect was not significant. When the arginine concentration was 20 mM, aging was completely suppressed up to 48 h of incubation. Similar effects of arginine were observed on the FUS-WT droplets (Fig. S4). These results indicate that arginine preserves the liquid properties of the droplets and slows the aging process. The liquid–solid transition, including fibrous aggregation, was suppressed for the proteins with arginine concentrations of 20 mM (Figs. 7b and S4b). These findings strongly support the idea that HP-LLPS formation, followed by the progressive increase in the intra- and inter-molecular interactions, is a probable pathway for the aberrant aggregation of FUS at atmospheric conditions.

### MD simulations

**Figure 8:**
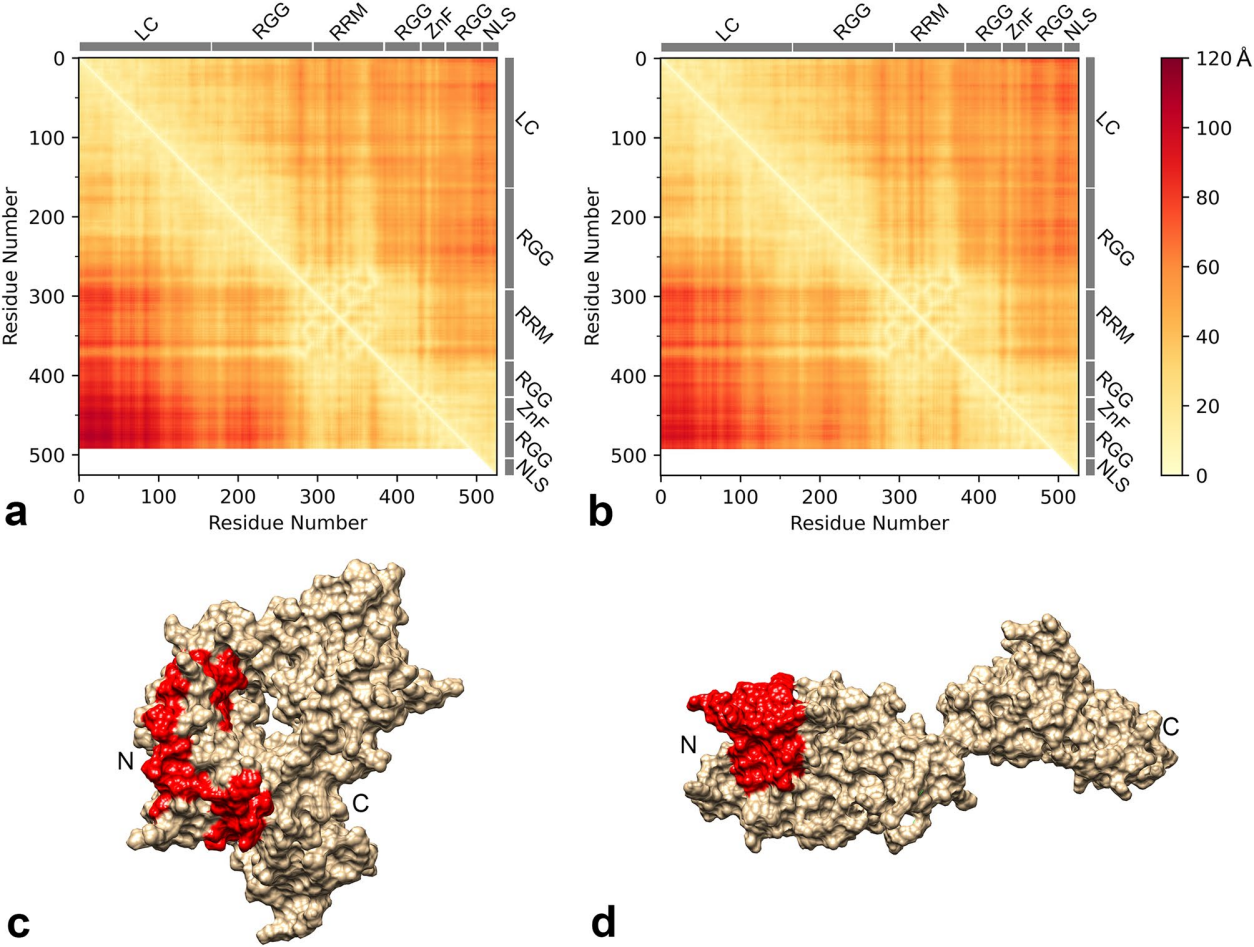
Molecular dynamics simulations of FUS-WT and R495X. (**a** and **b**) Distance maps between each residue at 1 bar and 3 kbar, respectively. Data for FUS-WT and R495X are presented in the top-right and bottom-left panels, respectively, in each panel. Low-complexity region, Arg-Gly-Gly region, RNA recognition motif, zinc finger region, and nuclear localization signal are depicted by LC, RGG, RRM, ZnF, and NLS, respectively, at the top and right sides of the panels. (**c** and **d**) MD simulation snapshots of FUS-WT and R495X, respectively, at 100 ns, shown by molecular surfaces. The fibril core (a.a. 39–95) in the LC region is indicated in red. N- and C-terminals are depicted by N and C, respectively.

We have shown that both FUS-WT and the disease-type variant R495X form two types of droplets and undergo an irreversible transition to β-sheet-containing structures, and structural transition is particularly accelerated in R495X. To date, it is difficult to experimentally capture the tertiary structure information of the proteins in their LLPS states. Instead, useful information can be obtained from an MD simulation of a protein in water. We performed 4 × 100 ns MD simulations of FUS-WT and R495X in water at 1 bar and 3 kbar at 300 K. Distance maps between each residue in WT and R495X at 1 bar are presented in the top-right and bottom-left panels, respectively, of Fig. 8a. Distance maps of similar formats at 3 kbar are presented in Fig. 8b. We found that the WT polypeptide chain was more frequently observed to be compact than that of R495X, regardless of pressure conditions. On the one hand, distances between the residues in the LC region and those in the C-terminal region were shorter in WT (< 75 Å) than in R495X (< 110 Å), probably because of the larger number of Tyr-Arg interactions, resulting in the compaction of the WT polypeptide chain. On the other hand, in R495X, the distances were markedly longer, probably because of the lack of the seven arginine residues in the C-terminal region. Note that the gyration radiuses of WT were 30 ± 4 and 31 ± 5 Å at 1 bar and 3 kbar, respectively, and those of R495X were 36 ± 2 and 33 ± 5 Å at 1 bar and 3 kbar, respectively (Fig. S5). Figure 8c and d show MD simulation snapshots of WT and R495X, respectively, at 100 ns and 1 bar. In WT, a part of the fibril core (a.a. 39–95) in the LC region^40^ either faces inward or steric hindrance, rendering it difficult for molecular interactions to occur; however, in R495X, the entire region is exposed. Such molecular conformations of the proteins were not substantially affected by pressure in terms of the distance map (Fig. 8b). Although these MD simulation results explain the structure in the mixed phase, the structural differences may influence intermolecular interaction in the droplets and irreversible conformational transitions.

## Discussion

The formation and disruption of LLPS are well-regulated in cells. Targeting the LLPS of proteins is a novel therapeutic strategy, as disruptions in LLPS regulation may be associated with neurodegenerative diseases. In the present study, we found that the disease variant R495X, which lacks 32 amino acid residues at its C-terminus, has two LLPS states in common with WT. The similarity between the *p–T* phase diagrams of the two proteins suggests that partial molar volume and enthalpy differences between 1-phase and each LLPS state are similar for both proteins. However, a comparison of the kinetic parameters of LLPS formation and disappearance obtained from the pressure-jump experiments showed that the two LLPS states of R495X formed and disappeared 2–threefold slower than those of the WT, i.e., 1.5–1.7-fold in LP-LLPS and 2–2.6-fold in HP-LLPS. In addition, the droplet aging accelerated by HP-LLPS was more pronounced in R495X than in the WT. These results imply that intermolecular interactions within the liquid droplets were not same between the two proteins but were expected to be stronger in R495X than in WT—differences are more pronounced in HP-LLPS. Alternatively, if the transition state theory applies to the formation and disappearance of each LLPS, the slower rates indicate that the transition states are more unstable in R495X than in the WT. The formation and disappearance of LP-LLPS were reversibly repeated in both WT and R495X. A large difference between the two proteins was clearly observed when the pressure-jump experiments were repeated between 2 and 3.5 kbar. Significant irreversibility was observed only in R495X, namely in the cycles of the formation and disappearance of HP-LLPS, whereas they were still reversible within 10 min of pressure-jump cycles in WT. Irreversibility has also been observed in WT when the time spent in the HP-LLPS state is increased to more than 30 min^28^. Microscopic observation at 1 bar also revealed that R495X formed a larger amount of irreversible aggregates than WT (Fig. 4c).

The thermodynamic interconversion between the 1-phase and the two LLPS states occurs in equilibrium, with the reversible formation and disappearance of the LLPS states. Moreover, interconversion between the two LLPS states reportedly occurs^26^. Under low-temperature conditions, LP-LLPS appeared to change continuously into HP-LLPS as the pressure increased, suggesting that HP-LLPS is rare but exists with LP-LLPS even under atmospheric pressure, regardless of solution conditions such as those *in cellula* and in vitro. These previous and current results support the notion that HP-LLPS develops irreversible properties and accelerates its transition to solid aggregates in physiological conditions^28^. It is believed that the probability of HP-LLPS droplets or the intermolecular interactions within the droplets, i.e., differences in the properties of the sequence, accelerate the transition from reversible droplets to solids. Because the absorbance and the *p*–*T* phase diagram are similar between WT and R495X, we may consider that the probability of HP-LLPS droplets is also similar at any temperature and pressure conditions between the two proteins. Therefore, the intermolecular interaction is a more plausible factor for droplet aging than the probability of HP-LLPS droplets in the case of FUS LLPS. MD simulations showed a difference in molecular conformation between WT and R495X. The average distances between the arginine-rich 35 residues in the C-terminal segments and the tyrosine-rich regions (i.e., LC region) were shorter in WT than in R495X, resulting in WT polypeptide chain compaction. Although the current MD simulations do not reflect the structure and dynamics of the polypeptide chain in the droplet, the conformational differences may lead to differences in the intermolecular interactions of the proteins in the LLPS states. We speculate that the intramolecular interactions between the LC and C-terminal regions suppress the time-dependent progress of intermolecular interactions and subsequent conversion of the fibril core into the β-sheet in WT, whereas the time-dependent molecular interaction and structural conversion could be promoted by R495X because of the exposure of the fibril core.

In addition, we discovered that ~ 20 mM arginine, dopamine, and pyrocatechol suppressed the formation of HP-LLPS more effectively than LP-LLPS in both WT^28^ and R495X and that even 1 mM of arginine delayed droplet aging (Fig. 6c). In fact, 20 mM arginine almost completely suppressed R495X aggregation over 48 h, although 2 mM arginine permitted a rapid increase in the number of aggregates after 24 h (Fig. 7b). Arginine is hydrophobic, aromatic, and cationic. Notably, arginine is the third most aromatic of the 20 amino acids after tryptophan and tyrosine^41^. Therefore, arginine inhibits the formation of HP-LLPS via hydrophobic, π–π, and cation–π interactions. Dopamine and pyrocatechol inhibited the HP-LLPS of R495X less effectively than arginine. Our findings demonstrate that guanidino and catechol (or polyphenol) groups act selectively on the HP-LLPS. In general, small-molecule compounds can be delivered into cells more easily than peptide or protein drugs. A plasma arginine concentration of 0.6 mM was achieved when rabbits and rats were treated with arginine orally^42^. Even if the intracellular concentration is sub-mM, the effect of delaying liquid–solid phase transition is expected to be sufficient. Kamagata et al. found that arginine and tyrosine had significant inhibitory effects on the thermal aggregation of FUS^43^. In addition, when FUS aggregates were induced by hydrogen peroxide in dopaminergic neurons, 0.3 mM arginine was found to suppress FUS aggregation. Therefore, we believe that administering arginine might delay disease onset and progression associated with ALS-causing FUS. Although it has been hypothesized that the physiological significance of FUS droplets and stress granules in cells is in RNA processing and the temporary storage of proteins in cells, these physiological functions should not be inhibited by drugs. Here, we suggest a novel concept for drug development targeting HP-LLPS that effectively decreases the aberrant aggregation of proteins with minimal impact on physiological functions. This concept is generally applicable to the liquid droplet–mediated aggregation pathway of proteins, regardless of post-translational modifications of proteins in cells, including methylation of arginine and phosphorylation of serine and threonine^31,44,45^.

## Conclusions

In this study, we reported the reversible formation of the two LLPS states and the irreversible liquid–solid transition of the ALS patient-type FUS variant, R495X. The liquid–solid phase transition was more accelerated in the HP-LLPS of R495X than that of the WT. Moreover, arginine suppressed the formation of HP-LLPS more selectively than that of LP-LLPS and delayed the aging of R495X droplets at atmospheric conditions. These findings strongly suggest that HP-LLPS is rare but exists with LP-LLPS even under atmospheric pressure and may explain why R495X readily forms cytoplasmic granules in neurons and develops a more aggressive disease phenotype. Thus, our findings may support the development of a novel approach to drug development by targeting HP-LLPS. However, the molecular-level differences in the internal structure of LLPS states are still largely unknown. Pressure perturbation is an effective approach for spectroscopic and microscopic observations of rare and aberrant HP-LLPS states. In addition, experiments using mammalian cells and ALS-model animals are needed to clarify the therapeutic effects of small molecules in preventing and treating ALS-causing FUS.

## Materials and methods

### Sample

FUS fused to a maltose-binding protein (MBP) containing a TEV cleavage site was expressed in BL21 (DE3) *Escherichia coli*^1^. After purification by affinity chromatography with amylose resin (New England Biolabs Japan Inc., Tokyo, Japan), the MBP domain was cleaved using His-tagged TEV protease to release the full-length FUS^1^. The FUS R495X variant lacking C-terminal residues (at 495–526) was also prepared via fusion with MBP containing the TEV cleavage site^26,28^. TEV protease was prepared as described previously^28^. TEV protease was added at a final concentration of 10 µg/mL to 5 µM MBP-FUS protein in a buffer containing 20 mM HEPES (pH 7.4), 150 mM NaCl, 20 µM Zn(OAc)_2_, and 2 mM DTT with 10% glycerol and incubated at 30 °C for more than 3 h. For fluorescence microscopy experiments, MBP-tagged FUS-WT fused to GFP at the C-terminus was also prepared. The MBP domain was cleaved using the His-tagged TEV protease according to the aforementioned protocol. GFP-fused FUS-WT was mixed with the WT or R495X variant at a concentration of 5% for fluorescence microscopy measurements.

### Temperature-scan UV–Vis spectroscopy

UV–Vis experiments were conducted between 10 and 40 °C using a V730 spectrophotometer (JASCO Co., Tokyo, Japan) with an automatic 8-position Peltier cell changer PAC-743 (JASCO Co.). Nitrogen gas was used to eliminate water vapor in the spectrophotometer. The turbidity (i.e., absorbance) of the solution was measured at 400 nm for a mixed solution of FUS (R495X or WT), MBP, and TEV. The cloud points were estimated using a drawing method^26^.

### High-pressure UV–Vis spectroscopy

High-pressure UV–Vis measurements were conducted using a V-730 spectrophotometer (JASCO Co.) and a PCI-500 pressure-resistant optical cell (Syn-Corporation Co., Kyoto, Japan) containing a quartz inner cell. The optical cell temperature was controlled using a water bath. A hand pump was connected to the cell through a stainless-steel tube. The temperature was maintained within ± 0.1 °C and pressure within ± 10 bar. Nitrogen gas was used to eliminate water vapor from the pressure-resistant optical cell of the spectrophotometer. Thereafter, the protein solution was stored in a quartz inner cell. The turbidity (i.e., absorbance) of the solution was measured at 400 nm for a mixed solution of FUS R495X, MBP, and TEV; the control value was obtained at atmospheric pressure using water at 25 °C. The absorbance of the solution was measured following the convergence of the change in absorbance (e.g., 3–15 min). The transition pressures and cloud points were estimated using a drawing method^26^. Pressure-jump experiments were conducted between 1 bar and 1.2 kbar and 2.0–3.5 kbar by manually operating the hand pump and valve within a few seconds to change pressure. Absorbance was measured at 0.5–1 s intervals with a response time of 0.24 s after the pressure jump.

### Fluorescence microscopy

An optical microscope (LV100ND; Nikon Co., Tokyo, Japan) with 20 × or 40 × objectives (S Plan Fluor; Nikon), an LED light source for fluorescence (D-LEDI; Nikon), and a fluorescence filter (GFP Basic C-FL; Nikon) were used to observe the liquid and solid condensates of FUS-WT and R495X. The temperature was maintained within ± 0.1 °C. FUS-WT and R495X, including a 5% volume of GFP-fused FUS-WT, were prepared at 5 µM at 30 °C (above the cloud point) and centrifuged at 30 °C for 3 min at 12,000 rpm (max 11,000 G) to remove the initial irreversible aggregates in the sample solution. A sample chamber with a thickness of approximately 0.2 mm was created by placing a doughnut-shaped spacer between the glass slide and cover glass. The sample was incubated at 10 °C to generate liquid droplets. For microscopic observations, a variable temperature plate (SCP-85; AS ONE, Osaka, Japan) was used to maintain a temperature of 10 or 30 °C. Image processing and analysis (e.g., particle counts) were conducted by ImageJ Fiji^46,47^ and Python.

ThT assays were performed on FUS-WT and R495X without GFP fusion. The final concentration of ThT was 5 µM in the sample solution. Fluorescence microscopy images were collected at 0 h, 12 h, 24 h, 36 h, and 48 h of incubation at 10 °C using the fluorescence filter (GFP Basic C-FL) to observe ThT fluorescence.

### MD simulation

MD simulations were conducted to investigate the structure and dynamics of FUS-WT and R495X. Crystal structures of the RNA recognition motif (PDB ID: 2LA6) and the zinc finger domain (PDB ID: 6G99) of FUS were used to develop initial coordinates of the proteins for MD simulations. The disordered regions of FUS were modeled as extended structures. By connecting these structured and disordered regions, the overall structures of the proteins were constructed. To equilibrate the structures, 500-ns MD simulations were performed with the implicit solvent, where the proteins were described using the AMBER14SB force field^48^ and Generalized Born energy^49^.

Subsequently, each protein was simulated in a water box. The simulation system contained a FUS molecule (WT or R495X) and 111,100 water molecules for WT and 158,756 for R495X in a dodecahedron box with 17-nm sides. Additionally, 16 and 12 chloride ions for WT and R495X, respectively, were added to the system to neutralize the charge. The protein and water molecules were described using the AMBER03w force field^50^ and TIP4P/2005 model^51^, respectively. Zinc and chloride ion models were obtained from literature^52^.

After energy minimization, constant-pressure and -temperature (NPT) MD simulations were performed at 1 bar or 3 kbar and 300 K for 0.1 ns to equilibrate the system. The production runs were performed for 100 ns, with four runs performed for each pressure with different initial velocities. The Parrinello–Rahman method^53^ was used to maintain pressure during the NPT simulation, and Langevin dynamics was used to maintain the temperature, with water viscosity set at 2 ps^−1^. The covalent bonds of hydrogen atoms in proteins were constrained using the LINCS method^54^, and the integration time step was 2.0 fs. These MD simulations were conducted using GROMACS 2020.6^55^. To evaluate residue–residue distance and the radius of gyration, 10–100-ns trajectories were used. C_b_–C_b_ distances were calculated for all residue pairs; however, C_a_ was used in the case of glycine.

### Statistics and reproducibility

Temperature-scan experiments were performed three times for R495X and five times for WT. A phase diagram was generated by performing multiple temperature- and pressure-scan experiments at the same protein concentration. The *p*–*T* phase diagram of LLPS is reproducible. Pressure-jump UV–Vis experiments were first conducted to optimize the pressure and temperature condition and then performed once with the optimized condition. High-pressure UV–Vis experiments were performed once as a function of arginine, dopamine, and pyrocatechol concentrations. Fluorescence microscope experiments were performed in triplicate for each experimental condition. ThT fluorescence image data for WT and R495X were collected from two and one experiment, respectively. 100 ns MD simulations were conducted four times for each pressure.

### Supplementary Information

Supplementary Figures.

Supplementary Information.

## Data Availability

All the data supporting this study are available in the paper and the supporting information. All numerical data sets are provided as a source data file. High-resolution microscopy images are available from the corresponding author upon request.

## References

[1] Yoshizawa T (2018). Nuclear import receptor inhibits phase separation of FUS through binding to multiple sites. Cell.

[2] Bracha D, Walls MT, Brangwynne CP (2019). Probing and engineering liquid-phase organelles. Nat. Biotechnol..

[3] Zbinden A, Perez-Berlanga M, De Rossi P, Polymenidou M (2020). Phase separation and neurodegenerative diseases: A disturbance in the force. Dev. Cell.

[4] Alberti S, Hyman AA (2021). Biomolecular condensates at the nexus of cellular stress, protein aggregation disease and ageing. Nat. Rev. Mol. Cell. Biol..

[5] Shapiro DM, Ney M, Eghtesadi SA, Chilkoti A (2021). Protein phase separation arising from intrinsic disorder: First-principles to bespoke applications. J. Phys. Chem. B.

[6] Kanaan NM, Hamel C, Grabinski T, Combs B (2020). Liquid–liquid phase separation induces pathogenic tau conformations in vitro. Nat. Commun..

[7] Berkeley RF, Kashefi M, Debelouchina GT (2021). Real-time observation of structure and dynamics during the liquid-to-solid transition of FUS LC. Biophys. J..

[8] Shuster SO, Lee JC (2022). Watching liquid droplets of TDP-43(CTD) age by Raman spectroscopy. J. Biol. Chem..

[9] Dec R, Jaworek MW, Dzwolak W, Winter R (2023). Liquid-droplet-mediated ATP-triggered amyloidogenic pathway of insulin-derived chimeric peptides: Unraveling the microscopic and molecular processes. J. Am. Chem. Soc..

[10] Cinar H, Fetahaj Z, Cinar S, Vernon RM, Chan HS, Winter R (2019). Temperature, hydrostatic pressure, and osmolyte effects on liquid-liquid phase separation in protein condensates: Physical chemistry and biological implications. Chem. Eur. J..

[11] Brangwynne CP (2009). Germline P granules are liquid droplets that localize by controlled dissolution/condensation. Science.

[12] Le Ferrand H, Duchamp M, Gabryelczyk B, Cai H, Miserez A (2019). Time-resolved observations of liquid–liquid phase separation at the nanoscale using in situ liquid transmission electron microscopy. J. Am. Chem. Soc..

[13] Shin Y, Berry J, Pannucci N, Haataja MP, Toettcher JE, Brangwynne CP (2017). Spatiotemporal control of intracellular phase transitions using light-activated optodroplets. Cell.

[14] Wang J (2018). A molecular grammar governing the driving forces for phase separation of prion-like RNA binding proteins. Cell.

[15] Wen J (2021). Conformational expansion of tau in condensates promotes irreversible aggregation. J. Am. Chem. Soc..

[16] Jawerth L (2020). Protein condensates as aging Maxwell fluids. Science.

[17] Murakami K, Kajimoto S, Shibata D, Kuroi K, Fujii F, Nakabayashi T (2021). Observation of liquid-liquid phase separation of ataxin-3 and quantitative evaluation of its concentration in a single droplet using Raman microscopy. Chem. Sci..

[18] Emmanouilidis L (2021). NMR and EPR reveal a compaction of the RNA-binding protein FUS upon droplet formation. Nat. Chem. Biol..

[19] Burke A, Janke AM, Rhine CL, Fawzi NL (2015). Residue-by-residue view of in vitro FUS granules that bind the c-terminal domain of RNA polymerase II. Mol. Cell.

[20] Ackermann BE, Debelouchina GT (2019). Heterochromatin Protein HP1α gelation dynamics revealed by solid-state NMR spectroscopy. Angew. Chem. Int. Ed..

[21] Bramham JE, Golovanov AP (2022). Temporal and spatial characterisation of protein liquid–liquid phase separation using NMR spectroscopy. Nat. Commun..

[22] Vance C (2013). ALS mutant FUS disrupts nuclear localization and sequesters wild-type FUS within cytoplasmic stress granules. Hum. Mol. Genet..

[23] Portz B, Lee BL, Shorter J (2021). FUS and TDP-43 phases in health and disease. Trends Biochem. Sci..

[24] Shiihashi G, Ito D, Yagi T, Nihei Y, Ebine T, Suzuki N (2016). Mislocated FUS is sufficient for gain-of-toxic-function amyotrophic lateral sclerosis phenotypes in mice. Brain.

[25] Wolozin B, Ivanov P (2019). Stress granules and neurodegeneration. Nat. Rev. Neurosci..

[26] Li S, Yoshizawa T, Yamazaki R, Fujiwara A, Kameda T, Kitahara R (2021). Pressure and temperature phase diagram for liquid-liquid phase separation of the RNA-binding protein fused in sarcoma. J. Phys. Chem. B.

[27] Patel A (2015). A liquid-to-solid phase transition of the ALS protein FUS accelerated by disease mutation. Cell.

[28] Li S (2022). Mechanism underlying liquid-to-solid phase transition in fused in sarcoma liquid droplets. Phys. Chem. Chem. Phys..

[29] Krainer G (2021). Reentrant liquid condensate phase of proteins is stabilized by hydrophobic and non-ionic interactions. Nat. Commun..

[30] Kitahara R (2021). Pressure-jump kinetics of liquid–liquid phase separation: Comparison of two different condensed phases of the RNA-binding protein, fused in sarcoma. J. Am. Chem. Soc..

[31] Kawahara D, Suzuki T, Nakaya T (2021). Cytoplasmic granule formation by FUS-R495X is attributable to arginine methylation in all Gly-rich, RGG1 and RGG2 domains. Genes Cells.

[32] Gonzalez A (2021). Mechanism of karyopherin-β2 binding and nuclear import of ALS variants FUS (P525L) and FUS (R495X). Sci. Rep..

[33] Nakaya T, Maragkakis M (2018). Amyotrophic lateral sclerosis associated FUS mutation shortens mitochondria and induces neurotoxicity. Sci. Rep..

[34] Waibel S (2013). Truncating mutations in FUS/TLS give rise to a more aggressive ALS-phenotype than missense mutations: A clinico-genetic study in Germany. Eur. J. Neurol..

[35] Waibel S, Neumann M, Rabe M, Meyer T, Ludolph AC (2010). Novel missense and truncating mutations in FUS/TLS in familial ALS. Neurology.

[36] Avrami M (1939). Kinetics of phase change I: General theory. J. Chem. Phys..

[37] Avrami M (1941). Granulation, phase change, and microstructure: Kinetics of phase change. III. J. Chem. Phys..

[38] Blazquez JS, Manchon-Gordon AF, Ipus JJ, Conde CF, Conde A (2018). On the use of JMAK theory to describe mechanical amorphization: A comparison between experiments, numerical solutions and simulations. Metals.

[39] Lelito J (2020). Crystallization kinetics analysis of the amorphouse Mg(72)Zn(24)Ca(4)alloy at the isothermal annealing temperature of 507 K. Materials.

[40] Murray DT (2017). Structure of FUS protein fibrils and its relevance to self-assembly and phase separation of low-complexity domains. Cell.

[41] Hirano A, Kameda T (2021). Aromaphilicity index of amino acids: Molecular dynamics simulations of the protein binding affinity for carbon nanomaterials. ACS Appl. Nano Mater..

[42] Morita M (2014). Oral supplementation with a combination of L-citrulline and L-arginine rapidly increases plasma L-arginine concentration and enhances NO bioavailability. Biochem. Biophys. Res. Commun..

[43] Kamagata K (2021). Characterization of design grammar of peptides for regulating liquid droplets and aggregates of FUS. Sci. Rep..

[44] Monahan Z (2017). Phosphorylation of the FUS low-complexity domain disrupts phase separation, aggregation, and toxicity. EMBO J..

[45] Qamar S (2018). FUS Phase separation is modulated by a molecular chaperone and methylation of arginine cation-π interactions. Cell.

[46] Schneider CA, Rasband WS, Eliceiri KW (2012). NIH image to ImageJ: 25 years of image analysis. Nat. Methods.

[47] Schindelin J (2012). Fiji: An open-source platform for biological-image analysis. Nat. Methods.

[48] Maier JA, Martinez C, Kasavajhala K, Wickstrom L, Hauser KE, Simmerling C (2015). ff14SB: Improving the accuracy of protein side chain and backbone parameters from ff99SB. J. Chem. Theory Comput..

[49] Mongan J, Simmerling C, McCammon JA, Case DA, Onufriev A (2007). Generalized Born model with a simple, robust molecular volume correction. J. Chem. Theory Comput..

[50] Best RB, Hummer G (2009). Optimized molecular dynamics force fields applied to the helix–coil transition of polypeptides. J. Phys. Chem. B.

[51] Abascal JLF, Vega C (2005). A general purpose model for the condensed phases of water: TIP4P/2005. J. Chem. Phys..

[52] Li P, Roberts BP, Chakravorty DK, Merz KM (2013). Rational design of particle mesh Ewald compatible Lennard–Jones parameters for + 2 metal cations in explicit solvent. J. Chem. Theory Comput..

[53] Parrinello M, Rahman A (1980). Crystal-structure and pair potentials: A molecular-dynamics study. Phys. Rev. Lett..

[54] Hess B, Bekker H, Berendsen HJC, Fraaije JGEM (1997). LINCS: A linear constraint solver for molecular simulations. J. Comput. Chem..

[55] Abraham MJ, Murtola T, Schulz R, Páll S, Smith JC, Hess B, Lindahl E (2015). GROMACS: High performance molecular simulations through multi-level parallelism from laptops to supercomputers. Software X.

