# Supplementary material for: An aberrant fused in sarcoma liquid droplet of amyotrophic lateral sclerosis pathological variant, R495X, accelerates liquid–solid phase transition
Source: Sci Rep. 2024 Apr 17;14:8914. doi: 10.1038/s41598-024-59604-4 (PMC11024109; doi:10.1038/s41598-024-59604-4)
Supplement: Supplementary file 1 — Supplementary Figures. [file 41598_2024_59604_MOESM1_ESM.pdf]

Supplementary Information

An aberrant fused in sarcoma liquid droplet of amyotrophic lateral sclerosis pathological variant, R495X, accelerates liquid–solid phase transition

Yutaro Shiramasa[a], *Ryu Yamamoto*[b], *Norika Kashiwagi*[b], *Fuka Sasaki*[b], *Sawaka Imai*[b], *Mikihito Ike*[b], *Soichiro Kitazawa*[b], *Tomoshi Kameda*[c], and *Ryo Kitahara*[a,b,*]

[a]Graduate School of Pharmacy, Ritsumeikan University, 1-1-1 Nojihigashi, Kusatsu, Shiga 525-8577, Japan

[b]College of Pharmaceutical Sciences, Ritsumeikan University, 1-1-1 Nojihigashi, Kusatsu, Shiga 525-8577, Japan

[c]Artificial Intelligence Research Center, National Institute of Advanced Industrial Science and Technology (AIST), 2-3-26, Aomi, Koto-ku, Tokyo 135-0064, Japan

Contents: Figures S1–S5

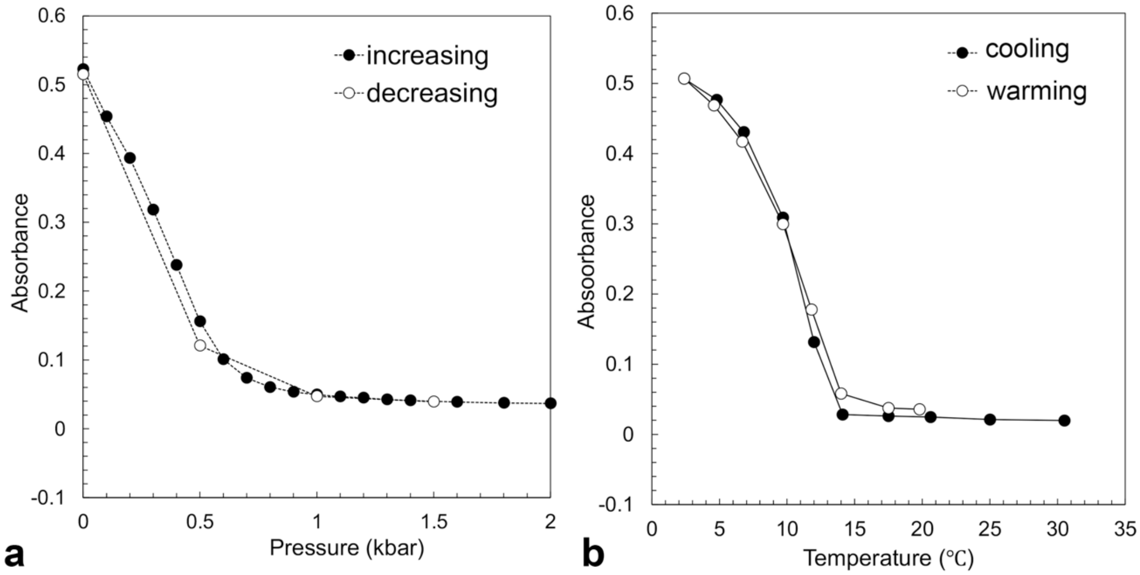

Figure S1. Pressure and temperature dependence of fused in sarcoma (FUS)-wild-type (WT) liquid–liquid phase separation (LLPS). (**a** and **b**) FUS-WT pressure-scan data at 15.6 °C and temperature-scan data at 3.1 kbar, respectively, are reproduced from the previous report[26].

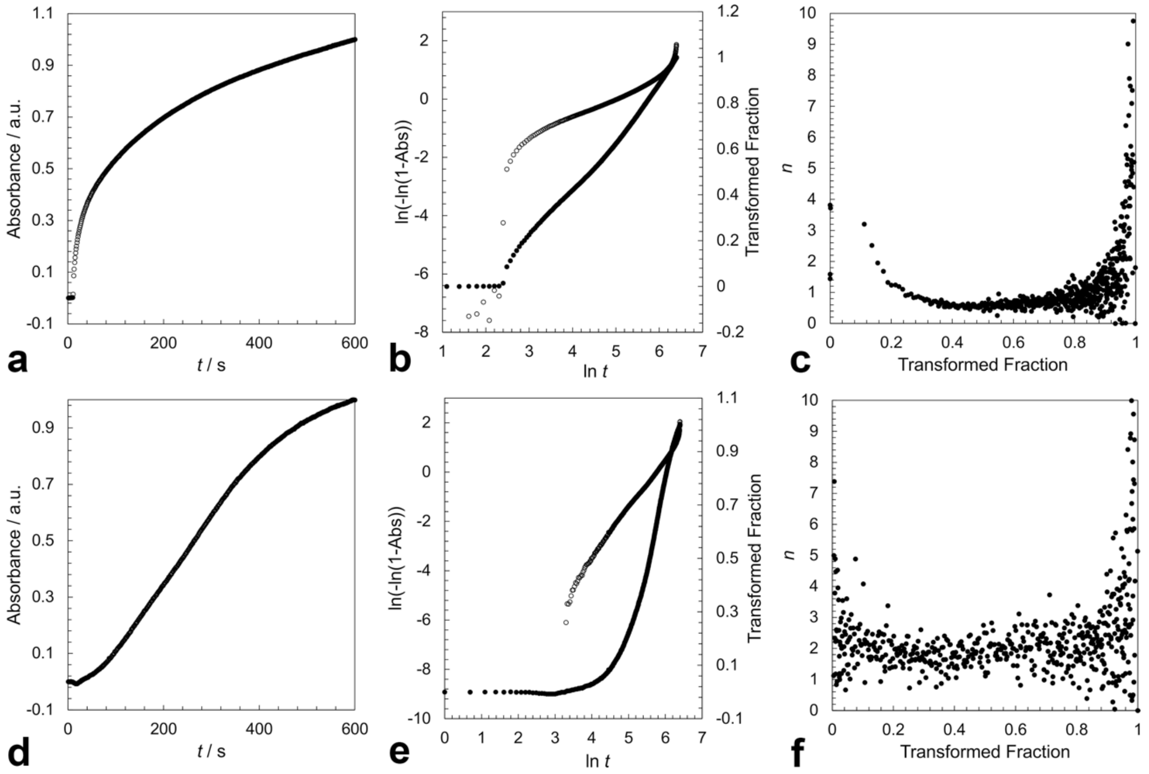

Figure S2. Kinetic analysis of liquid–liquid phase separation (LLPS) formation using the JMAK equation. (**a**) Time-dependent increase in normalized absorbance, corresponding to LP-LLPS formation, as pressure jumps from 1.2 kbar to 1 bar at 9.5 °C. (**b**) Dependence of ln [-ln(1-Abs)] (left scale, open circles) and the fraction transformed into the LP-LLPS state (right scale, closed circles) on ln *t*. (**c**) Avramin exponent *n* versus the transformed fraction, LP-LLPS. (**d**) Time-dependent increase in normalized absorbance, corresponding to HP-LLPS formation, as pressure jumps from 2.0 kbar to 3.5 bar at 9.4 °C. (**e**) Dependence of ln [-ln(1-Abs)] (left scale, open circles) and the fraction transformed into the HP-LLPS state (right scale, closed circles) on ln *t*. (**f**) Avramin exponent *n* versus the transformed fraction into HP-LLPS.

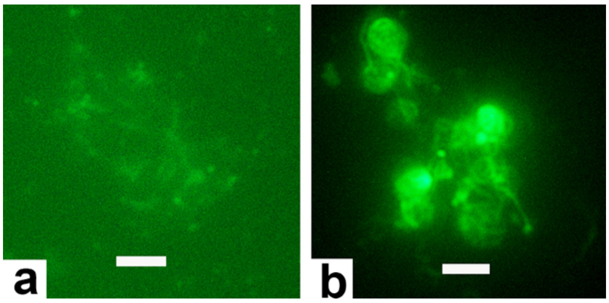

Figure S3. Fibrous aggregates of fused in sarcoma (FUS)-wild-type (WT) observed using fluorescence microscopy. (**a**) Fluorescence images of GFP-fused FUS observed when heated to 30 °C after 24 h of incubation at 10 °C. (**b**) ThT fluorescence images of FUS observed at 24 °C after 36 h of incubation at 24 °C. White bars = 10 μm.

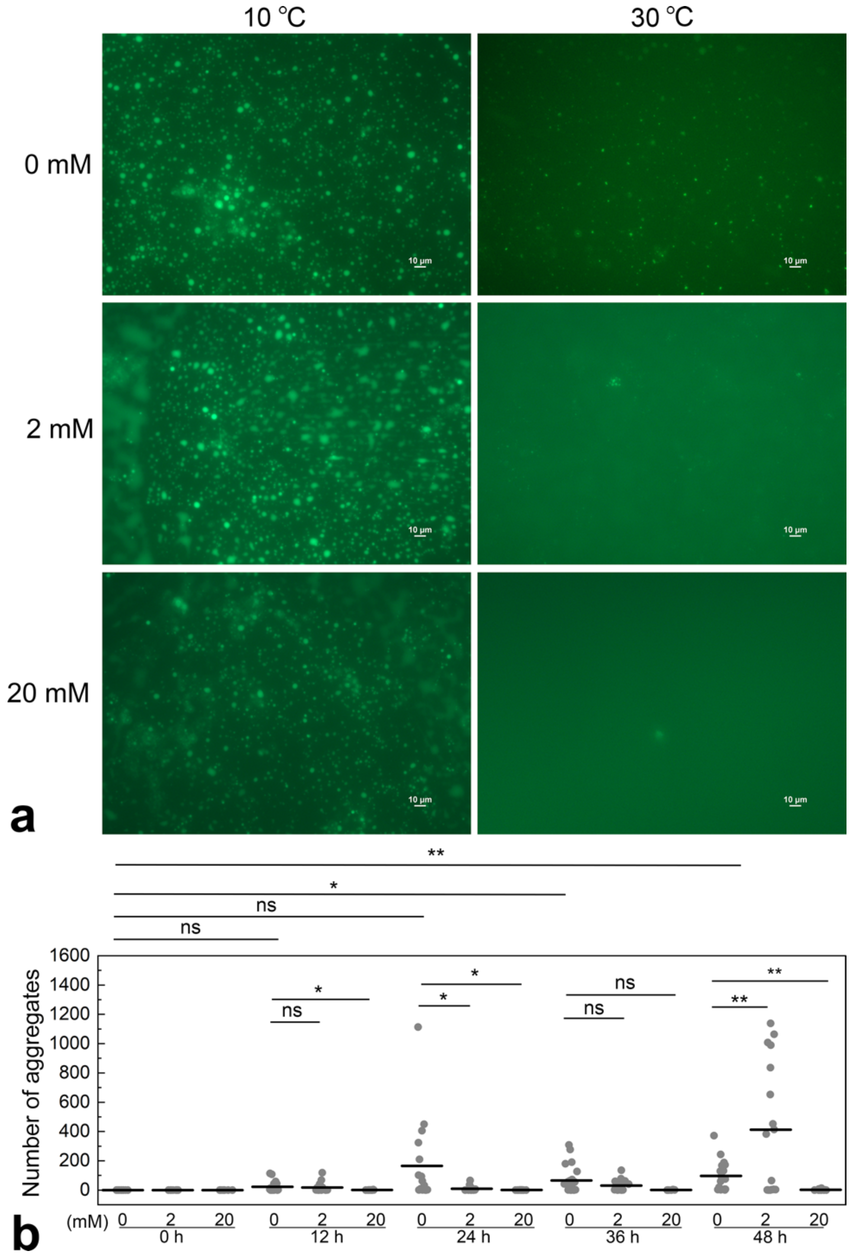

Figure S4. Arginine extends the reversible property of wild-type (WT) droplets. (**a**) Fluorescence microscopy images of WT (5 μM) droplets with different arginine concentrations (0–20 mM) obtained at 10 °C (left) and 30 °C (right) after 36 h incubation at 10 °C. Scale bar = 10 μm. The contrast and brightness of the images obtained at 30 °C were adjusted using Adobe Photoshop 2023 (Adobe, Mountain View, CA, USA). (**b**) The number of remaining droplets when heated to 30 °C after 12–48 h of incubation at 10 °C. Mean of particle counts were calculated using 6–23 microscopic images for each condition. * $P < 0.05$, ** $P < 0.01$, and ns=not significant using Student's t-test.

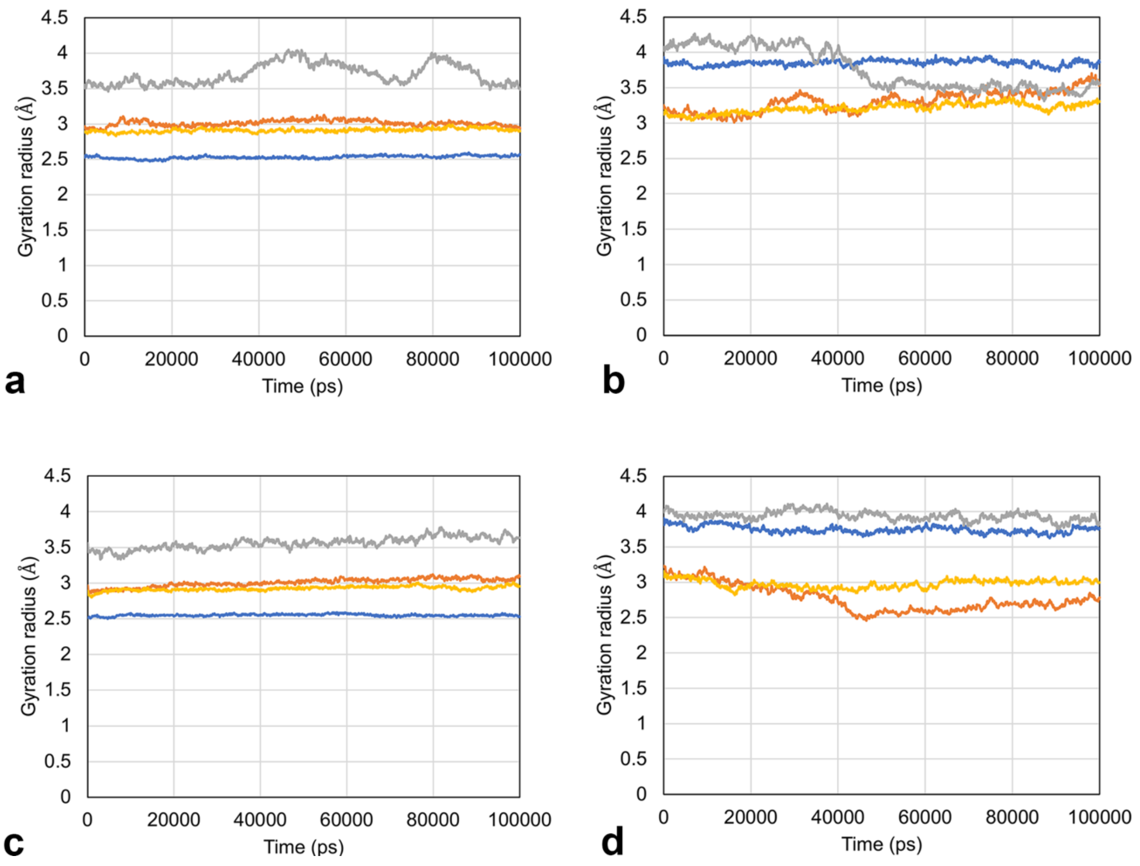

Figure S5. The radius of gyration estimated using molecular dynamics simulations. (**a**) Wild-type (WT) at 1 bar. (**b**) WT at 3 kbar. (**c**) R495X at 1 bar. (**d**) R495X at 3 kbar. Four independent results are represented by different colors.
